# Mitochondrial encephalomyopathy caused by a novel ACAD9 mutation: a case report

**DOI:** 10.3389/fnhum.2026.1828023

**Published:** 2026-06-11

**Authors:** Shuo Li, Yijun Li, Yonghua Chen

**Affiliations:** 1 The First Clinical Medical College of Anhui University of Chinese Medicine, The First Affiliated Hospital of Anhui University of Chinese Medicine, Hefei, Anhui, China; 2 Shuguang Anhui Hospital Affiliated to Shanghai University of Traditional Chinese Medicine (The First Affiliated Hospital West District of Anhui University of Chinese Medicine), Hefei, Anhui, China

**Keywords:** ACAD9, ataxia, case report, complex I deficiency, encephalomyopathy, mitochondrial disease

## Abstract

**Background** A 27-year-old male with perinatal hypoxia presented with global developmental delay, progressive hearing loss, ataxia, dysarthria, and intellectual disability. Whole-exome sequencing revealed compound heterozygous ACAD9 variants: c.456del (p.Ile153Serfs*46) and c.869G > A (p.Gly290Glu). Brain MRI showed bilateral cerebellar atrophy and a prominent cisterna magna. OCT confirmed optic atrophy. The diagnosis of mitochondrial encephalomyopathy (complex I deficiency type 20) was established. This report expands the known genetic spectrum associated with mitochondrial encephalomyopathy and underscores the critical role of genomic sequencing in diagnosing atypical, slowly progressive multisystem disorders.

## Introduction

Mitochondrial diseases encompass a heterogeneous group of disorders characterized by dysfunction of the mitochondrial oxidative phosphorylation system, with an estimated prevalence of approximately 1 in 4,300 individuals (Gorman et al., 2016). These conditions may manifest at any age and can affect virtually any organ system, with a particular predilection for tissues exhibiting high energy requirements, including the brain, heart, and skeletal muscle (Gorman et al., 2016). Consequently, encephalomyopathic presentations frequently emerge, encompassing developmental delay, seizures, stroke-like episodes, and sensorineural hearing loss (Lancaster and Graham, 2023). Specific mitochondrial syndromes, such as myoclonic epilepsy with ragged-red fibers (MERRF), exhibit characteristic features including myoclonus, epilepsy, and ataxia, which help differentiate them from other mitochondrial disorders (Finsterer, 2025).

Mitochondrial complex I (NADH: ubiquinone oxidoreductase) deficiency represents the most common respiratory chain defect associated with early-onset fatal encephalomyopathy. Complex I constitutes the largest of the five oxidative phosphorylation complexes, comprising 45 structural subunits; among these, 14 serve catalytic functions while the remaining 31 contribute to complex assembly, stability, and functional regulation (Leslie et al., 2016). Although numerous molecular defects have been identified in both mitochondrial and nuclear structural subunits as well as assembly factors, the molecular etiology remains elusive in many affected individuals. “Whole mitochondrial exome” sequencing—involving deep sequencing of the complete mitochondrial genome alongside coding exons of the thousands of nuclear genes encoding the mitochondrial proteome—has proven invaluable for identifying novel genetic variants associated with oxidative phosphorylation defects during infancy (Leslie et al., 2016; Repp et al., 2018).

Acyl-CoA dehydrogenase family member 9 (ACAD9) is a critical assembly factor for mitochondrial complex I. Biallelic pathogenic variants in ACAD9 cause complex I deficiency, a condition with highly variable clinical presentations (Leslie et al., 2016). Due to its rarity and phenotypic variability, ACAD9-related disease is often diagnosed late, particularly in patients with slow, progressive courses. Herein, we report a 27-year-old male with a novel compound heterozygous ACAD9 mutation, whose diagnosis was delayed for decades, and provide a comprehensive literature review.

## Case description

A 27-year-old right-handed male presented to our neurology clinic with complaints of progressively worsening gait unsteadiness and dysarthria. His medical history included perinatal hypoxia requiring neonatal care. At approximately 4 months of age, his family noted delayed responsiveness and hearing impairment. Subsequent developmental milestones were characterized by delayed attainment of independent standing, an unsteady gait, slurred speech, progressive hearing deterioration, blurred vision, intellectual disability relative to age-matched peers, slowed cognitive processing, and involuntary tremors involving the head and bilateral upper extremities. He had previously sought medical evaluation at multiple institutions, where investigations suggested “cerebellar hypoplasia.” There was no documented history of seizures, stroke-like episodes, or cardiomyopathy. Family history was significant for a younger sister who died at 17 years of age with a similar clinical phenotype.

Physical Examination: Neurological evaluation at our institution revealed multisystem involvement including dysarthria, nystagmus, a wide-based ataxic gait, and dysmetria on finger-to-nose testing. Intellectual function was diminished compared to peers, with slowed responsiveness. Involuntary tremors of the head and bilateral upper extremities were observed. Hearing impairment was confirmed by abnormal auditory evoked potentials. Visual complaints included blurred vision, and ophthalmological examination revealed bilateral ptosis.

**Figure 1:**
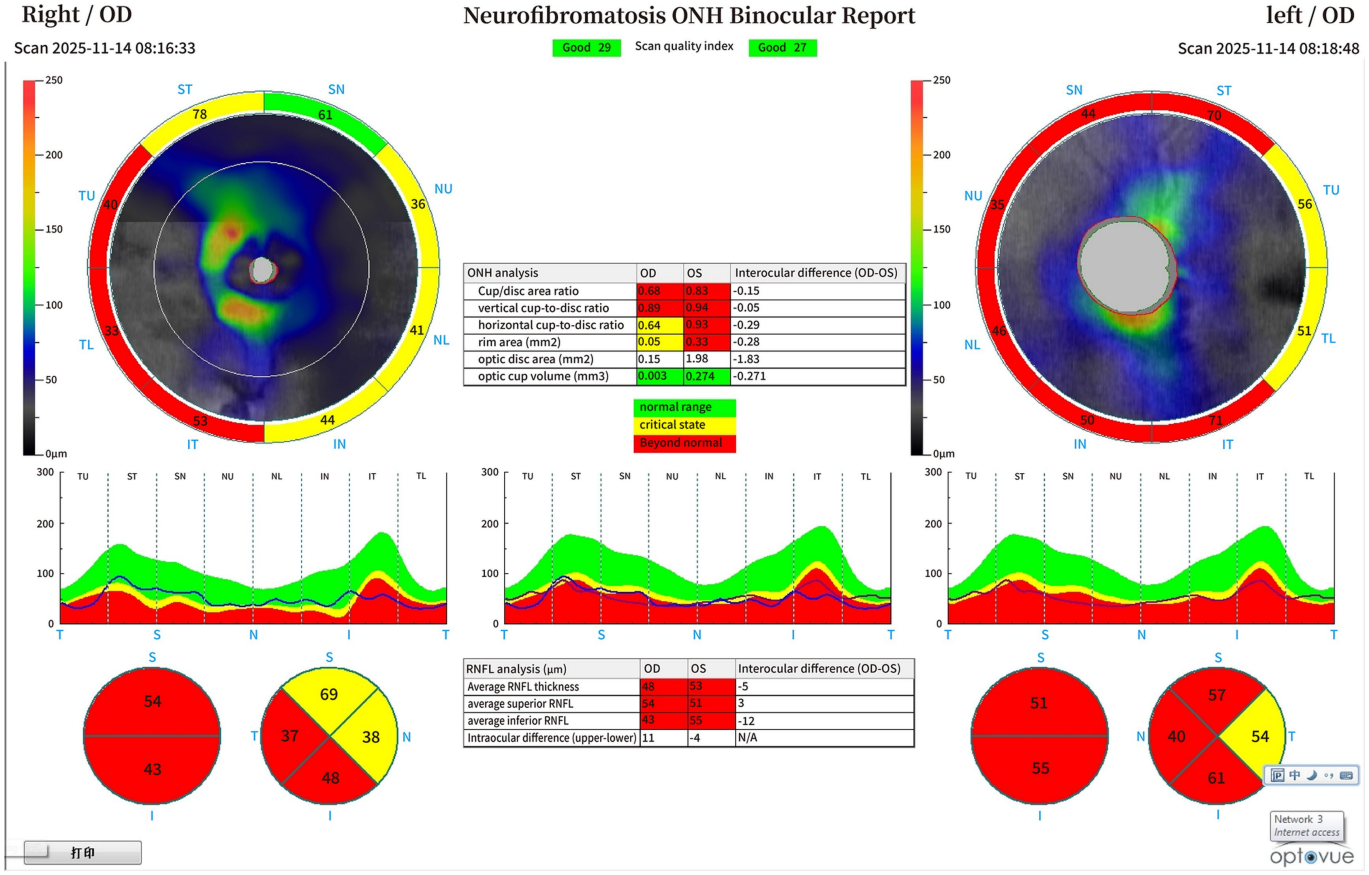
Optical coherence tomography. Optical coherence tomography demonstrates bilateral thinning of the peripapillary retinal nerve fiber layer (RNFL). Mean RNFL thickness measures 48 μm in the right eye and 53 μm in the left eye, both significantly below normal reference ranges. Optic disc area and rim area are reduced with correspondingly increased cup-to-disc ratios, consistent with optic atrophy.

Neuro-ophthalmological Assessment: Funduscopic examination demonstrated bilateral optic disc pallor. Optical coherence tomography (OCT) confirmed diffuse thinning of the peripapillary retinal nerve fiber layer (RNFL), with mean RNFL thickness measuring 48 μm in the right eye and 53 μm in the left eye. Optic disc area and rim area were reduced, with correspondingly increased cup-to-disc ratios, consistent with bilateral optic atrophy Figure 1.

Laboratory Findings: Serial laboratory evaluations revealed persistent hyperlactatemia, with plasma lactate levels of 5.06 mmol/L and 6.28 mmol/L on separate occasions (reference range: 0.5–2.2 mmol/L). Cerebrospinal fluid (CSF) examination showed an elevated lactate level of 2.78 mmol/L (reference range: 1.1–2.4 mmol/L), with no other significant abnormalities. Serum creatine kinase levels were within normal limits (95 U/L, 90 U/L, and 99 U/L; reference range: 20–200 U/L), arguing against significant myopathic involvement. These biochemical findings are consistent with mitochondrial complex I deficiency.

**Figure 2:**
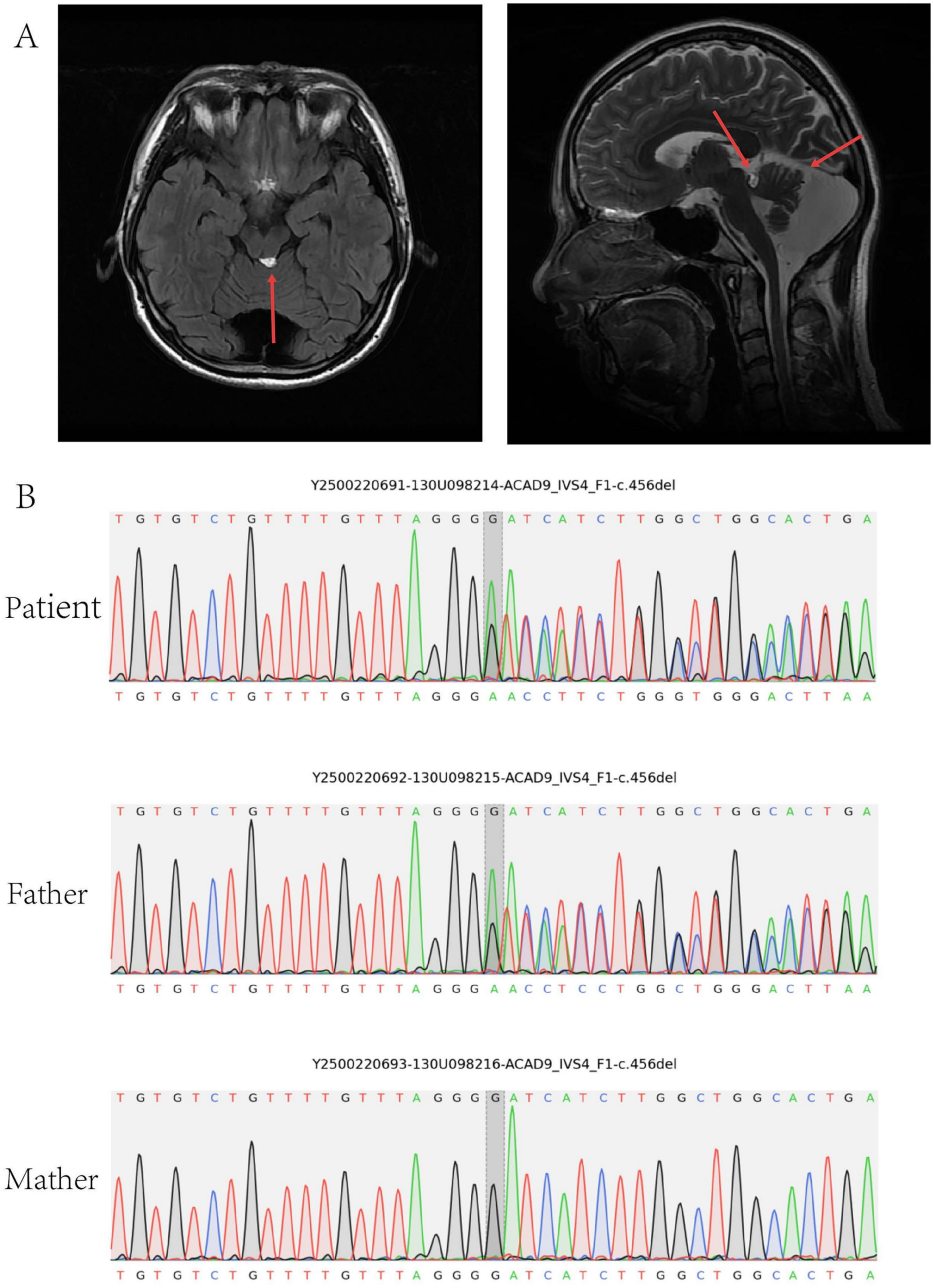
Neuroimaging and genetic features. **(A)** Axial T2-weighted or FLAIR MRI demonstrates bilateral cerebellar hemisphere atrophy (arrows) with marked enlargement of the cisterna magna. A Quadrigeminal cistern lipoma is visibled. **(B)** Sanger sequencing confirms compound heterozygous ACAD9 mutations. The proband carries both c.456del (frameshift, producing overlapping peaks following the deletion site indicated by arrow) and c.869G > A (missense). The father is heterozygous for c.456del; the mother is heterozygous for c.869G > A.

Neuroimaging: Cranial magnetic resonance imaging (MRI) with susceptibility-weighted imaging (SWI) and diffusion-weighted imaging (DWI) demonstrated marked enlargement of the cisterna magna, bilateral reduction in cerebellar hemisphere volume, and imaging features suggestive of a lipoma within the quadrigeminal cistern Figure 2A. These findings are consistent with neuroradiological features associated with mitochondrial encephalomyopathy (Fan et al., 2021).

Genetic Analysis: Whole-exome sequencing was performed on the proband and both parents using samples collected on the third day after admission, soon after the clinical suspicion of mitochondrial encephalomyopathy was raised. Regarding the deceased younger sister, who died at age 17 with a similar neurological phenotype, no cardiac abnormalities had been documented during her lifetime. However, neither autopsy nor genetic testing was performed, and it remains unknown whether she carried the same ACAD9 mutations. The patient harbored compound heterozygous variants in the ACAD9 gene (transcript NM_014049.5): a paternally inherited frameshift variant c.456del (p.Ile153Serfs*46) and a maternally inherited missense variant c.869G > A (p.Gly290Glu) Figure 2B. According to ACMG/AMP guidelines, the c.456del (p. Ile153Serfs*46) frameshift variant was classified as likely pathogenic (evidence codes: PVS1, PM2_Supporting). The c.869G > A (p. Gly290Glu) missense variant was classified as a variant of uncertain significance (evidence codes: PM2_Supporting, PM3, PP3_Moderate). Integration of the clinical phenotype, biochemical profile, and inheritance pattern consistent with autosomal recessive transmission established the diagnosis of mitochondrial encephalomyopathy due to ACAD9 mutation (complex I deficiency type 20).

Treatment and Follow-up: The patient was initiated on coenzyme Q10 supplementation at a daily dose of 30 mg (Shi et al., 2025), along with high-dose riboflavin (150 mg daily). To objectively assess treatment response, the Scale for the Assessment and Rating of Ataxia (SARA) was administered before and after supplementation. The total SARA score improved from 25 at initial admission to 14 at follow-up admission, representing a reduction of 11 points. Specifically, scores for items 1 (gait and stance), 5 (finger chase, mean of both sides), and 6 (nose-finger test, mean of both sides) each decreased by 2 points, while all remaining items (sitting, speech disturbance, fast alternating hand movements, heel-shin slide, etc.) each decreased by 1 point. Clinically, the patient showed marked improvements in gait stability, nystagmus, mental status, and psychomotor slowing. Activities of daily living, including independent walking, sitting balance, and feeding, were substantially enhanced according to family reports. Although serial blood lactate levels were not reassessed after treatment owing to the patient’s limited financial resources, the observed 11-point reduction in the SARA total score provides objective and reproducible evidence of clinical efficacy. The patient was counseled to avoid medications potentially impairing mitochondrial function, including valproate and statins. During subsequent follow-up, his clinical condition remained relatively stable.

## Discussion

This case illuminates the diagnostic challenges inherent in nuclear-encoded mitochondrial diseases, particularly among adults presenting with slowly progressive, multisystem neurodegeneration. The patient’s clinical phenotype—encompassing chronic cerebellar ataxia, sensorineural hearing loss, ophthalmoparesis (ptosis), peripheral neuropathy, cognitive decline, and persistent hyperlactatemia—aligns closely with mitochondrial encephalomyopathy (Fan et al., 2021). The documented optic nerve involvement, confirmed by OCT, further substantiates the multisystem nature of energy-dependent tissue damage. Although the patient lacked defining features of well-characterized mitochondrial syndromes such as MELAS (stroke-like episodes) or MERRF (myoclonus and epilepsy), the overall clinical presentation falls within the broad spectrum of mitochondrial disease. Definitive diagnosis was achieved through whole-exome sequencing, which identified compound heterozygous pathogenic variants in ACAD9.

ACAD9 serves as a critical assembly factor for mitochondrial complex I; its deficiency impairs oxidative phosphorylation, resulting in diminished cellular energy production (Leslie et al., 2016). The genetic findings correlate precisely with the family history: the patient’s affected sister likely harbored identical biallelic mutations, consistent with autosomal recessive inheritance. The c.456del (p.Ile153Serfs*46) variant is novel and predicted to induce frameshift with premature protein truncation, thereby expanding the genotypic spectrum of ACAD9-related disease. The c.869G > A (p.Gly290Glu) missense variant has been previously associated with complex I deficiency (Leslie et al., 2016).

Neuroimaging in this case revealed bilateral cerebellar atrophy, a finding commonly associated with ACAD9 deficiency, and additionally, a lipoma located in the quadrigeminal cistern. Typical neuroradiological features of ACAD9-related disorders include cerebellar and brainstem atrophy, white matter hyperintensities, and basal ganglia abnormalities, whereas intracranial lipomas have not been previously recognized as a component of this condition. The quadrigeminal cistern lipoma is therefore a novel observation that expands the radiological spectrum of ACAD9 deficiency.

The clinical relevance of this lipoma warrants consideration. The quadrigeminal cistern houses the superior and inferior colliculi, which are involved in visual and auditory processing, respectively. While the patient’s visual impairment and hearing loss are primarily attributable to mitochondrial optic neuropathy (confirmed by OCT) and sensorineural damage, the presence of a lipoma in this location could theoretically exert a mass effect on the tectal region, potentially modulating or exacerbating the clinical phenotype. Although most intracranial lipomas are incidental and asymptomatic, larger or strategically located lesions may affect adjacent neural structures. Thus, this finding not only expands the imaging phenotype of ACAD9 deficiency but also highlights the need to consider structural intracranial abnormalities as possible modifiers of neurological manifestations in mitochondrial disorders. Future case series should pay attention to the presence of such lipomas to determine whether this association is coincidental or reflects an unrecognized developmental vulnerability in ACAD9-related disease.

Several clinically relevant observations emerge from this case. First, persistent hyperlactatemia in patients with unexplained progressive neurological symptoms constitutes a powerful biomarker warranting consideration of mitochondrial disorders (Ching et al., 2013). Second, mitochondrial diseases should remain within the differential diagnosis for adolescents and adults presenting with complex multisystem involvement, even in the absence of acute, classic presentations (Gorman et al., 2016; Lancaster and Graham, 2023). Third, comprehensive ophthalmological evaluation, including OCT, may reveal subclinical optic neuropathy and provide additional evidence supporting multisystem disease (Watson-Fargie et al., 2025). Fourth, genomic sequencing—particularly whole-exome or “whole mitochondrial exome” approaches—is indispensable for confirming diagnosis, enabling accurate genetic counseling, and potentially guiding therapeutic strategies (Leslie et al., 2016; Repp et al., 2018; Luan and Liang, 2026). The present case further suggests that ACAD9 gene screening should be incorporated into diagnostic algorithms for unexplained ataxia accompanied by elevated lactate levels.

The relatively late onset of symptoms in this patient—clinical recognition in early childhood with a slowly progressive course into adulthood—contrasts with the classic infantile presentations of ACAD9 deficiency, which often involve fatal cardiomyopathy and lactic acidosis within the first year of life. This is likely explained by the patient’s genotype: compound heterozygosity of a truncating variant (c.456del, p.Ile153Serfs*46) and a missense variant (c.869G > A, p.Gly290Glu). A large cohort study reported that no individual with biallelic loss-of-function mutations survived, suggesting such a combination is incompatible with postnatal survival (Repp et al., 2018). The presence of at least one missense allele may be critical for survival, potentially retaining partial ACAD9 function, which is further supported by reports of individuals with missense variants who survived into adulthood with predominantly neurological involvement (Garone et al., 2013; Garone et al., 2013). Notably, both affected siblings in this family lacked cardiac abnormalities, suggesting that these specific mutations may preferentially impair neurological tissues while relatively sparing cardiac function. This observation aligns with the hypothesis that certain missense variants allow sufficient residual complex I activity in cardiomyocytes, pointing toward a predominantly encephalomyopathic subtype of ACAD9 deficiency. Together, these findings extend the recognized phenotypic continuum of ACAD9-related disorders—from fatal infantile encephalocardiomyopathy to slowly progressive, late-onset encephalomyopathy without cardiac involvement (Repp et al., 2018; Garone et al., 2013; Garone et al., 2013). No functional studies have been performed on the p. Gly290Glu variant identified in this patient; future in vitro investigations would be valuable to determine its residual enzymatic activity and tissue-specific effects.

Currently, no curative treatment exists for ACAD9 deficiency. Management focuses on symptomatic support and metabolic modulation. High-dose riboflavin (150 mg daily) has been shown to improve muscle strength and reduce plasma lactate and alanine levels in patients with ACAD9 deficiency, likely due to its role as a precursor for flavin cofactors that enhance residual complex I assembly and function (Schrank et al., 2017). Coenzyme Q10 supplementation aims to support residual mitochondrial function (Shi et al., 2025)^.^ Riboflavin has been demonstrated to enhance complex I activity in patient-derived fibroblasts and may improve clinical outcomes in some affected individuals (Repp et al., 2018); consequently, riboflavin supplementation could be considered as adjunctive therapy. Avoidance of medications with known mitochondrial toxicity, including valproate, represents a cornerstone of management (Koenig et al., 2016).

## Conclusion

We report a case of mitochondrial encephalomyopathy resulting from a novel compound heterozygous ACAD9 mutation. This case highlights a distinctive clinical phenotype characterized by progressive cerebellar ataxia, sensorineural hearing loss, visual impairment due to optic atrophy, and cognitive decline. Neuroimaging revealed bilateral cerebellar atrophy and, notably, a lipoma in the quadrigeminal cistern—an imaging finding not previously emphasized in association with ACAD9-related disorders. These observations expand the recognized clinical and radiological spectrum associated with ACAD9 mutations and may serve as valuable references for clinicians considering this diagnosis. This case further underscores the critical importance of genomic sequencing in diagnosing atypical, slowly progressive multisystem conditions. Enhanced awareness of such phenotypic presentations may facilitate earlier diagnosis and enable appropriate genetic counseling for affected families.

## Data Availability

The original contributions presented in the study are included in the article/supplementary material, further inquiries can be directed to the corresponding author.
